# Publisher Correction: Greater trophic diversity of soil animal communities under agricultural land use and tropical climate

**DOI:** 10.1038/s41559-026-03061-x

**Published:** 2026-04-01

**Authors:** Zheng Zhou, Nico Eisenhauer, Andrew D. Barnes, Melanie M. Pollierer, Malte Jochum, Ingo Grass, Yan Zhang, Ulrich Brose, Fujio Hyodo, Nicole Scheunemann, Olaf Schmidt, Yuanyuan Huang, Bernhard Klarner, Anton A. Goncharov, Alena Krause, Daniil Korobushkin, Anastasia Gorbunova, Ilya I. Lyubechanskii, Sergey M. Tsurikov, Julia Seeber, Michael Steinwandter, Vladimir A. Zryanin, Oksana L. Rozanova, Winda Ika Susanti, Felicity V. Crotty, Di Ajeng Prameswari, Zhipeng Li, Carol Melody, Zhijing Xie, Xue Pan, Donghui Wu, Mark Maraun, Katerina Sam, Alexei V. Tiunov, Stefan Scheu, Anton Potapov

**Affiliations:** 1 Animal Ecology, University of Göttingen, Göttingen, Germany; 2 Ecology of Tropical Agricultural Systems, University of Hohenheim, Stuttgart, Germany; 3 German Centre for Integrative Biodiversity Research (iDiv), Leipzig, Germany; 4 Institute of Biology, Leipzig University, Leipzig, Germany; 5 Te Aka Mātuatua - School of Science, University of Waikato, Hamilton, New Zealand; 6 Julius Kühn Institute, Federal Research Centre for Cultivated Plants, Institute for Forest Protection, Quedlinburg, Germany; 7 Department of Global Change Ecology, Biocenter, University of Würzburg, Würzburg, Germany; 8 Center for Biodiversity and Integrative Taxonomy (KomBioTa), University of Hohenheim, Stuttgart, Germany; 9 Institute of Biodiversity, Friedrich-Schiller-University Jena, Jena, Germany; 10 Faculty of Environmental, Life, Natural Science and Technology, Okayama University, Okayama, Japan; 11 Senckenberg Museum of Natural History Görlitz, Görlitz, Germany; 12 UCD School of Agriculture and Food Science Centre, University College Dublin, Dublin, Ireland; 13 A.N. Severtsov Institute of Ecology and Evolution, Russian Academy of Sciences, Moscow, Russia; 14 UCD School of Biology and Environmental Science, University College Dublin, Dublin, Ireland; 15 Institute of Systematics and Ecology of Animals of Siberian Branch of Russian Academy of Sciences (ISEA SB RAS), Novosibirsk, Russia; 16 Department of Natural Sciences, Novosibirsk State University, Novosibirsk, Russia; 17 Institute for Alpine Environment, Eurac Research, Bozen, Italy; 18 Universität Innsbruck, Department of Ecology, Innsbruck, Austria; 19 Lobachevsky State University, Nizhny Novgorod, Russia; 20 Global Land Team, Ricardo Energy and Environment, Didcot, UK; 21 Key Laboratory of Urban Environment and Health, Institute of Urban Environment, Chinese Academy of Sciences, Xiamen, China; 22 Key Laboratory of Vegetation Ecology, Ministry of Education, Northeast Normal University, Changchun, China; 23 Biology Centre, Czech Academy of Sciences, Institute of Entomology, České Budějovice, Czech Republic; 24 Faculty of Science, University of South Bohemia, České Budějovice, Czech Republic; 25 Southern Branch, Joint Russian-Vietnamese Tropical Science and Technology Research Center, Ho Chi Minh City, Vietnam; 26 Centre of Biodiversity and Sustainable Land Use, University of Göttingen, Göttingen, Germany; 27 International Institute Zittau, TUD Dresden University of Technology, Dresden, Germany

**Keywords:** Stable isotope analysis, Macroecology

Correction to: *Nature Ecology & Evolution* 10.1038/s41559-026-03014-4, published online 16 March 2026.

In the version of the article initially published, the *x* axis of Fig. 2c was “Variation of δ^15^ N” but should have been “Variation of δ^13^ C”. This error has now been corrected in the HTML and PDF versions of the article.

