# Supplementary material for: Greater trophic diversity of soil animal communities under agricultural land use and tropical climate
Source: Nat Ecol Evol. 2026 Mar 16;10(4):700–11. doi: 10.1038/s41559-026-03014-4 (PMC13076220; doi:10.1038/s41559-026-03014-4)
Supplement: Supplementary file 1 — Supplementary Figs. 1–13 and Tables 1–10. [file 41559_2026_3014_MOESM1_ESM.pdf]

nature ecology & evolution

Supplementary information https://doi.org/10.1038/s41559-026-03014-4

Greater trophic diversity of soil animal communities under agricultural land use and tropical climate

In the format provided by the authors and unedited

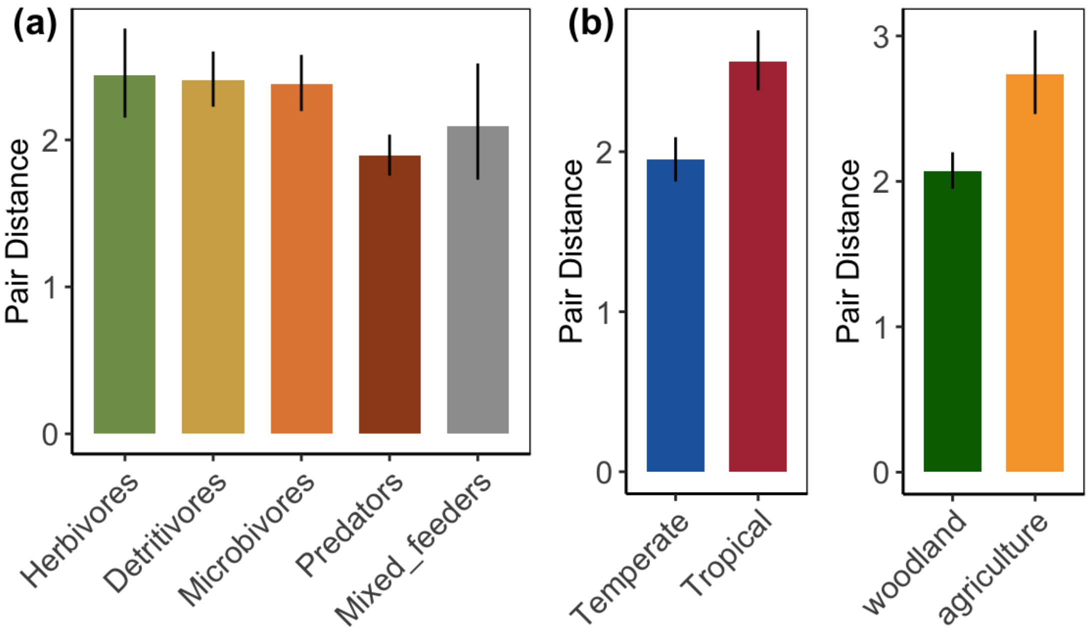

Fig. S1. Trophic dissimilarity (mean pairwise stable isotopic distance among the trophic-position centroids) of taxa in each functional group (a) and their variation between climatic regions and land-use systems (b).

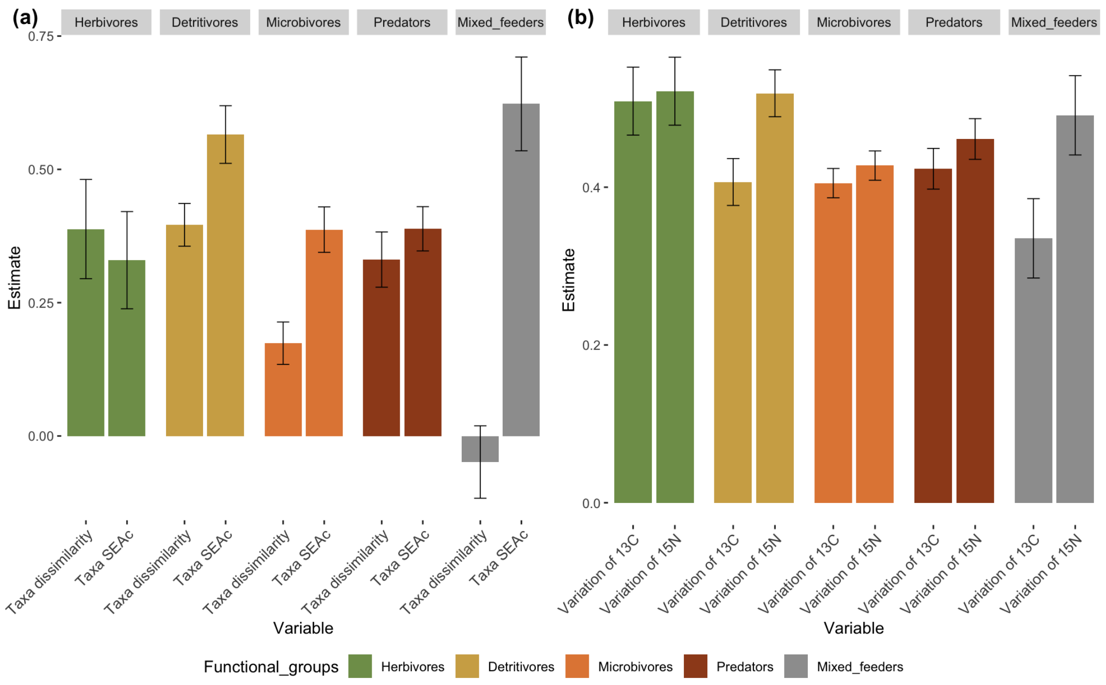

Fig. S2. The estimated value of the coefficient for (a) taxa SEAc and taxa dissimilarity in different functional groups of soil animals and (b) variation in $\delta^{13}C$ and $\delta^{15}N$ values to SEAc of different functional groups of soil animals.

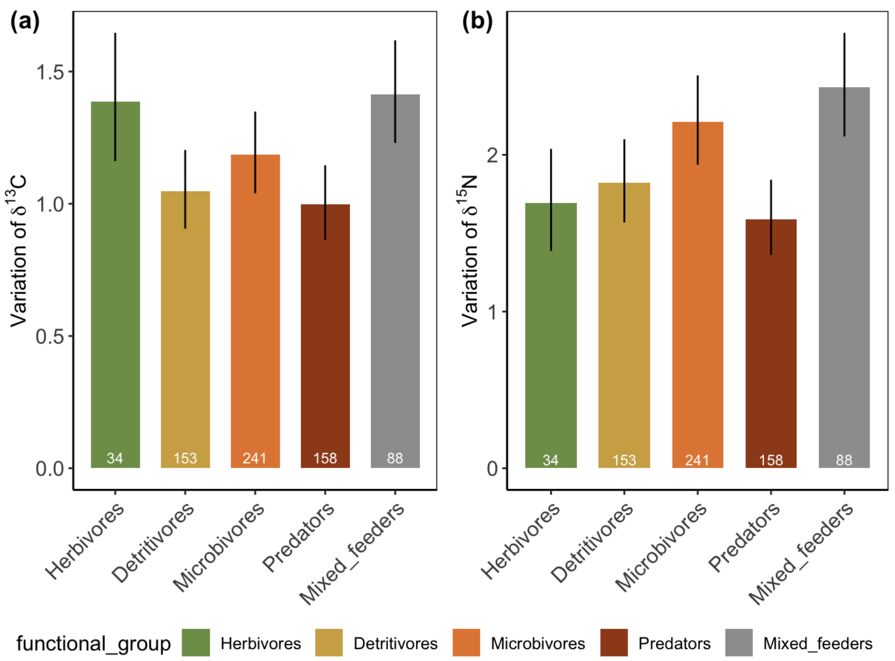

Fig. S3. Site-based variation of δ^{13}C (a) and δ^{15}N (b) in different functional groups, average values and 95% confidence interval, the number in the bar indicate the number of replicated sites.

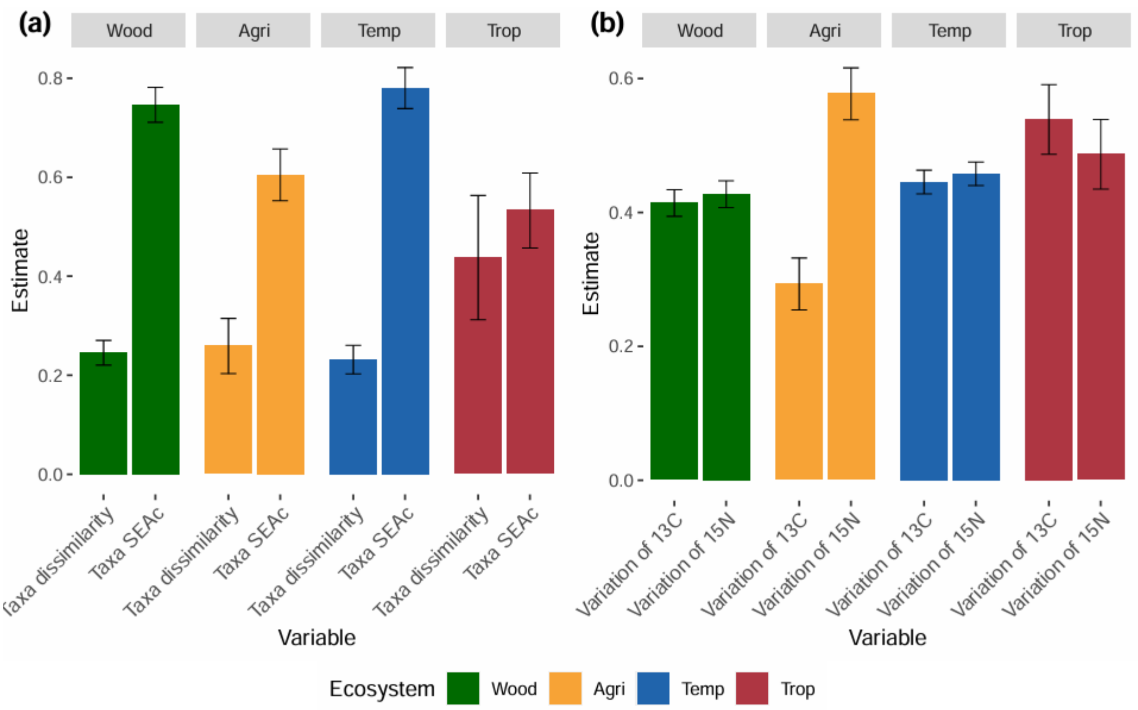

Fig. S4 The estimated value of the coefficient for (a) SEAc and taxa dissimilarity (b) Variation of ^{13}C and ^{15}N to estimate their contribution to SEAc of different land use systems (woodland, agricultural systems) and climatic regions (temperate, tropical regions).

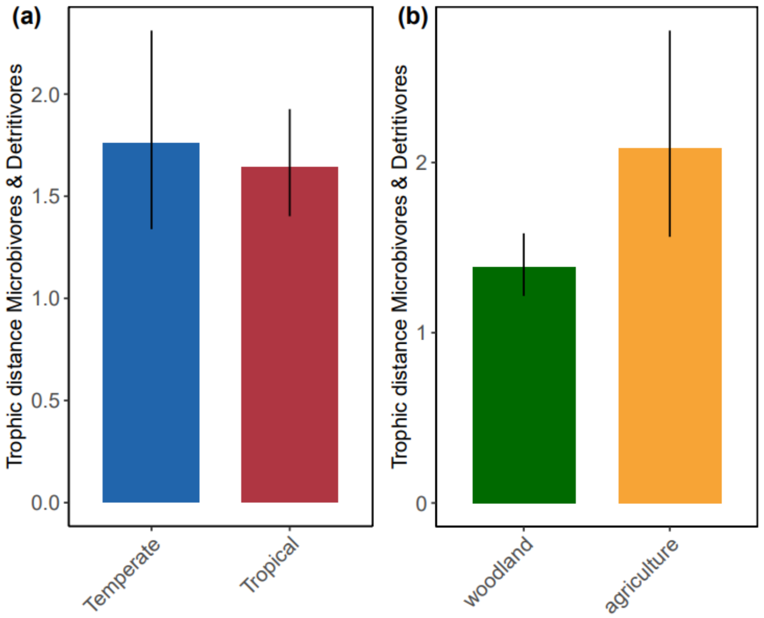

Fig. S5 Trophic dissimilarity of microbivores and detritivores between climatic regions (a) and land-use systems (b).

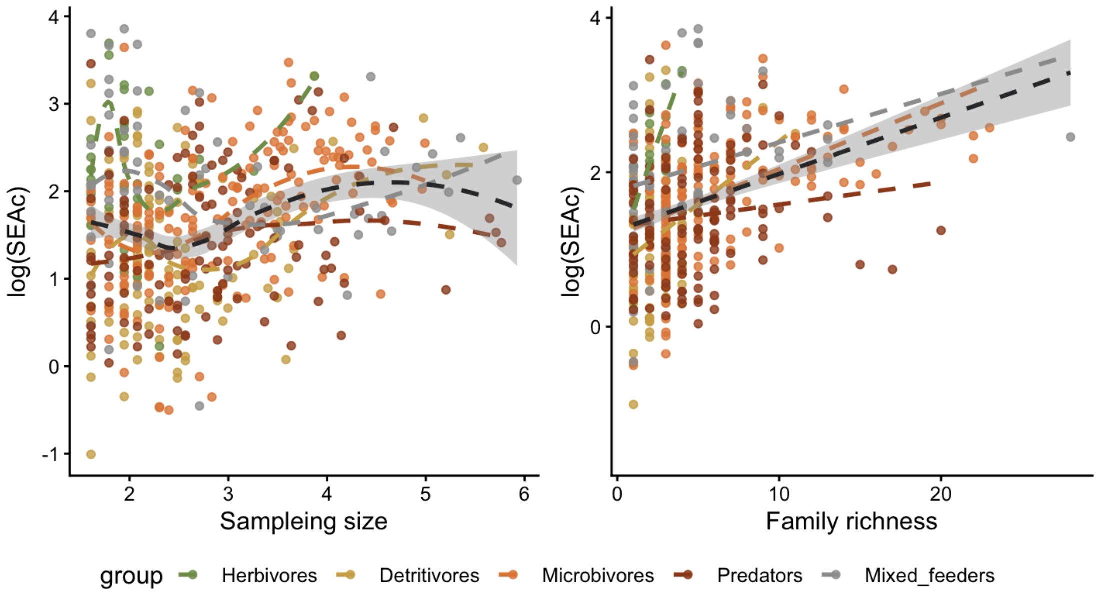

Fig. S6. The relation between sampling size and family richness with log-transformed SEAc (trophic diversity) of functional groups.

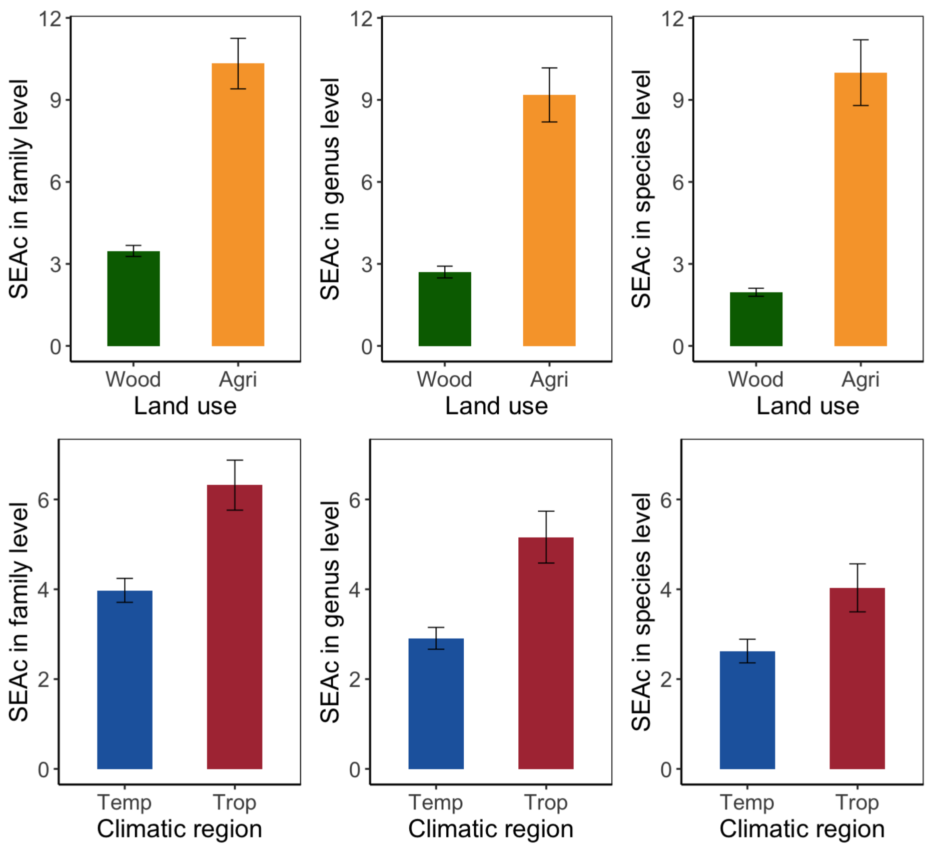

Fig. S7 The SEAc across different taxonomic levels in different land-use systems (woodland, agricultural systems) and climatic regions (temperate, tropical), means ± standard error.

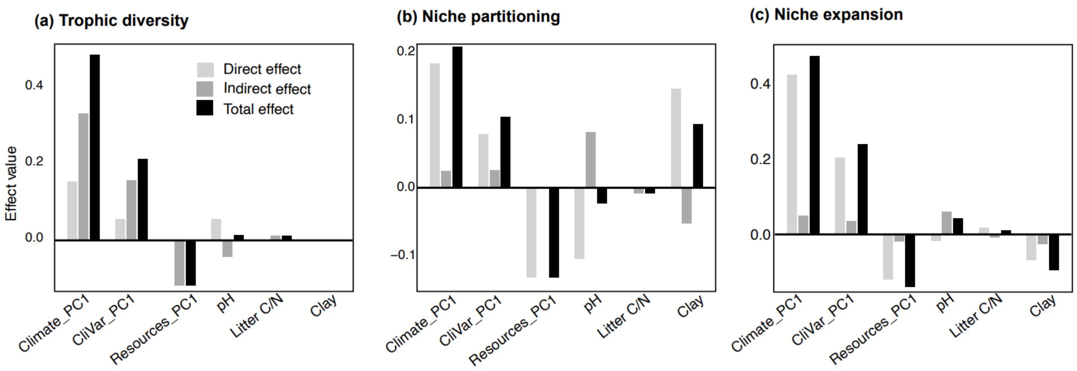

Fig. S8. Effects of environmental predictors for trophic diversity, niche partitioning and niche expansion of soil animals. Direct effects are indicated by light grey bars and indirect effects are indicated by dark grey bars and total effects are indicated by black bars.

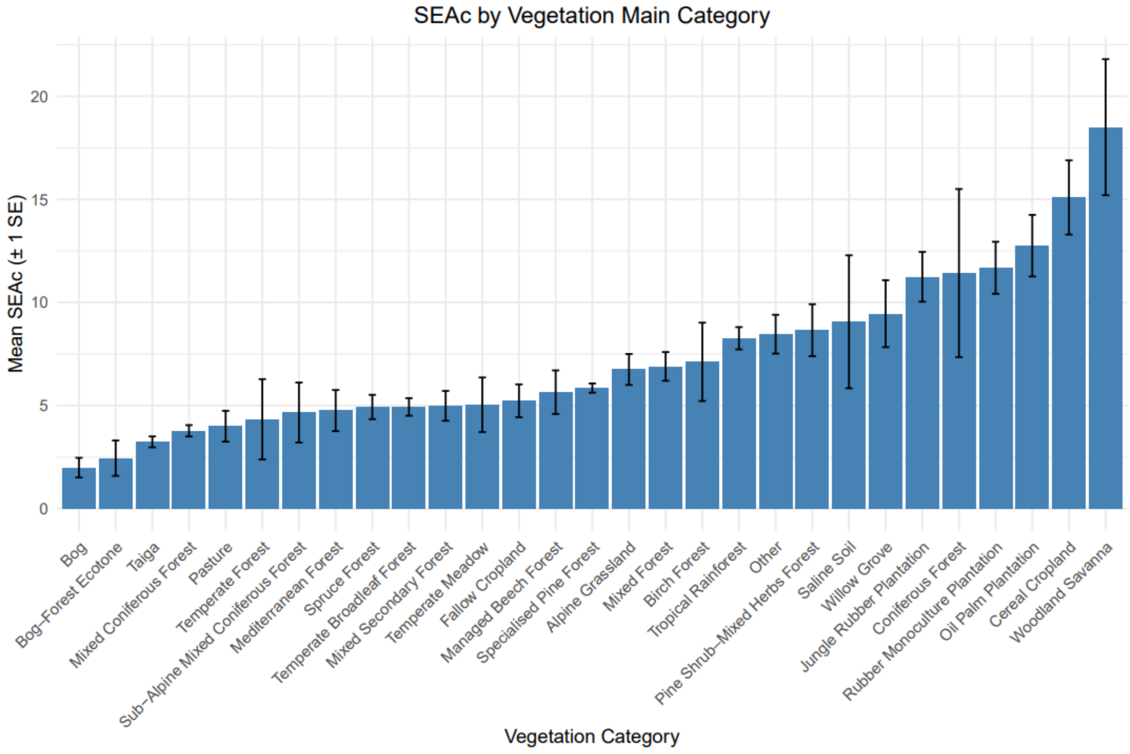

Fig. S9. The trophic diversity of soil animals (mean SEAc across functional groups) across 29 vegetation types, means ± standard error, reflecting different land-use and climatic contexts.

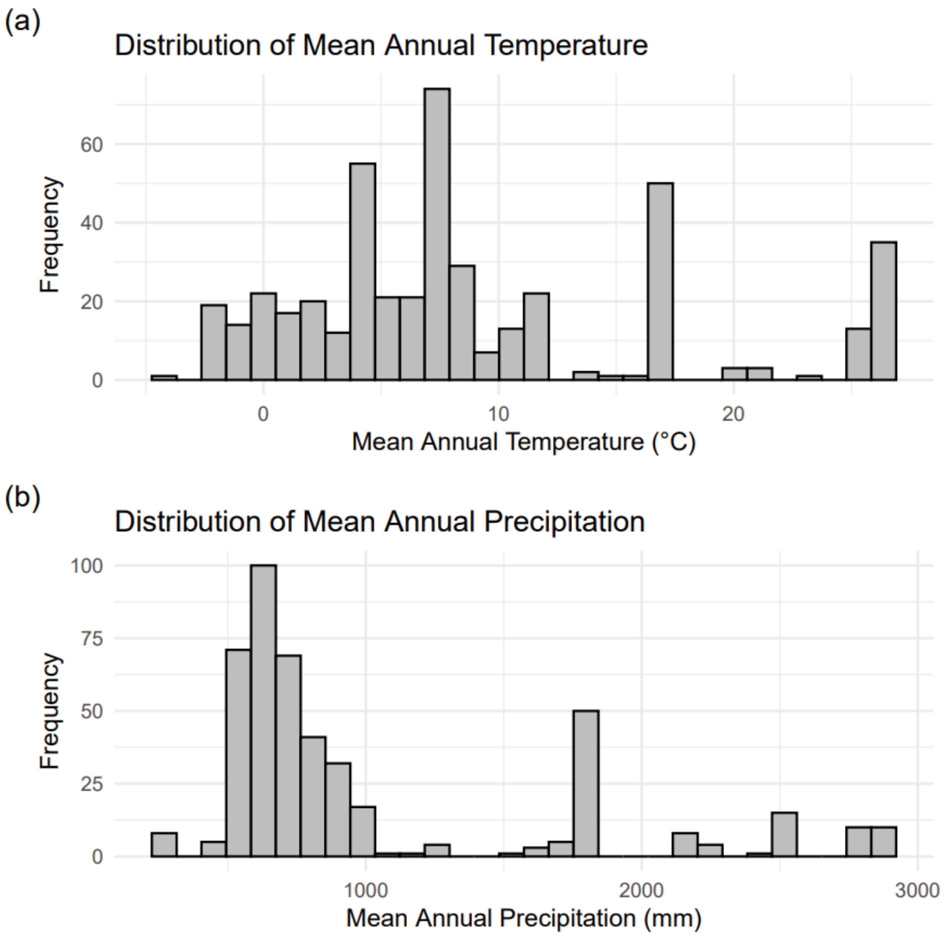

Fig. S10. Histograms showing the distribution of mean annual temperature and precipitation across unique sampling sites. (a) Frequency distribution of mean annual temperature (MAT; °C). (b) Frequency distribution of mean annual precipitation (MAP; mm). Data are based on one record per site.

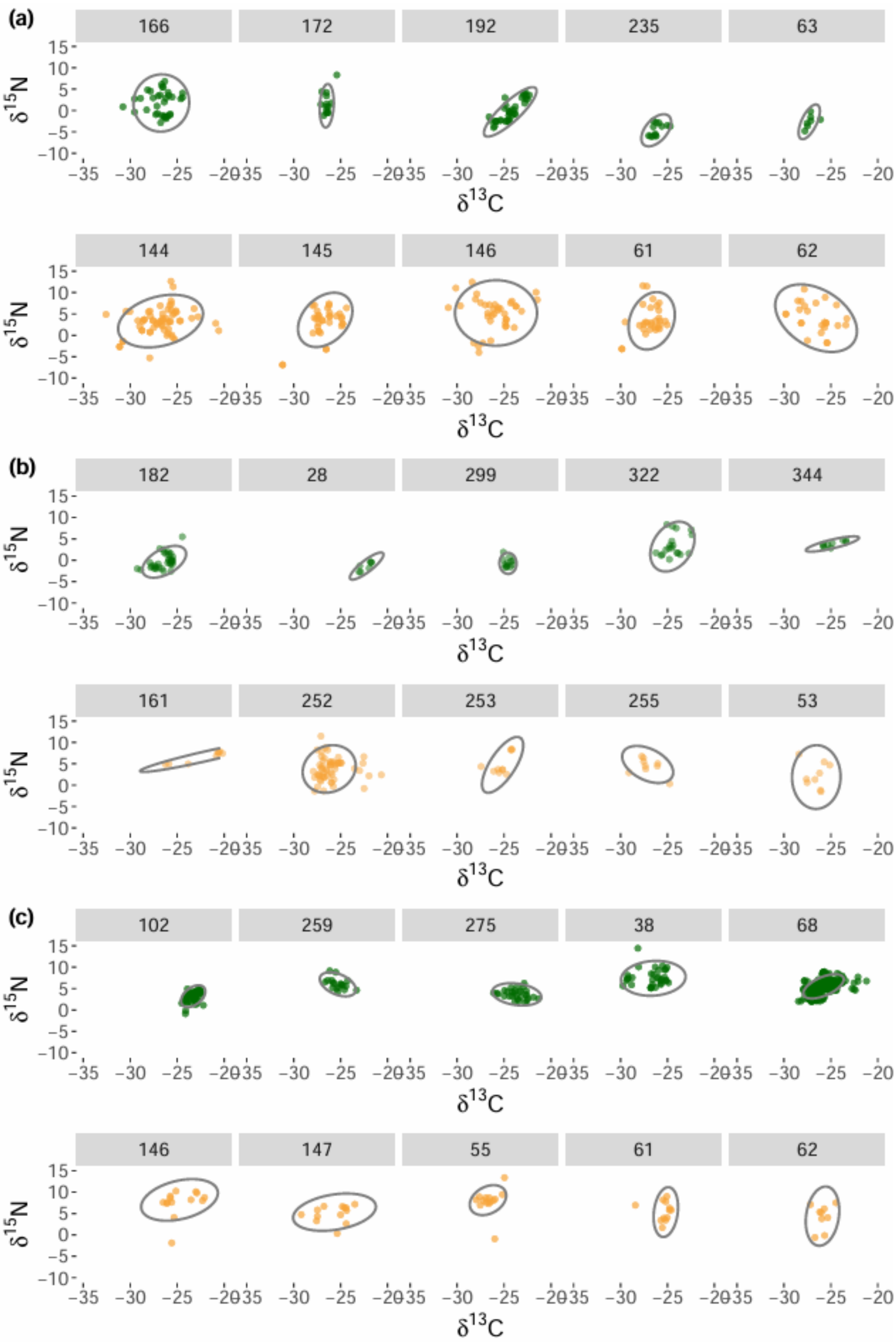

Fig. S11 Visualization of SEAc of (a) detritivores, (b) microbivores and (c) predators for randomly picked five sites in woodland and agricultural systems. Each dot represents one individual in woodland (green dots) or agriculture (yellow dots). The panel numbers indicate the site ID (reference to the dataset).

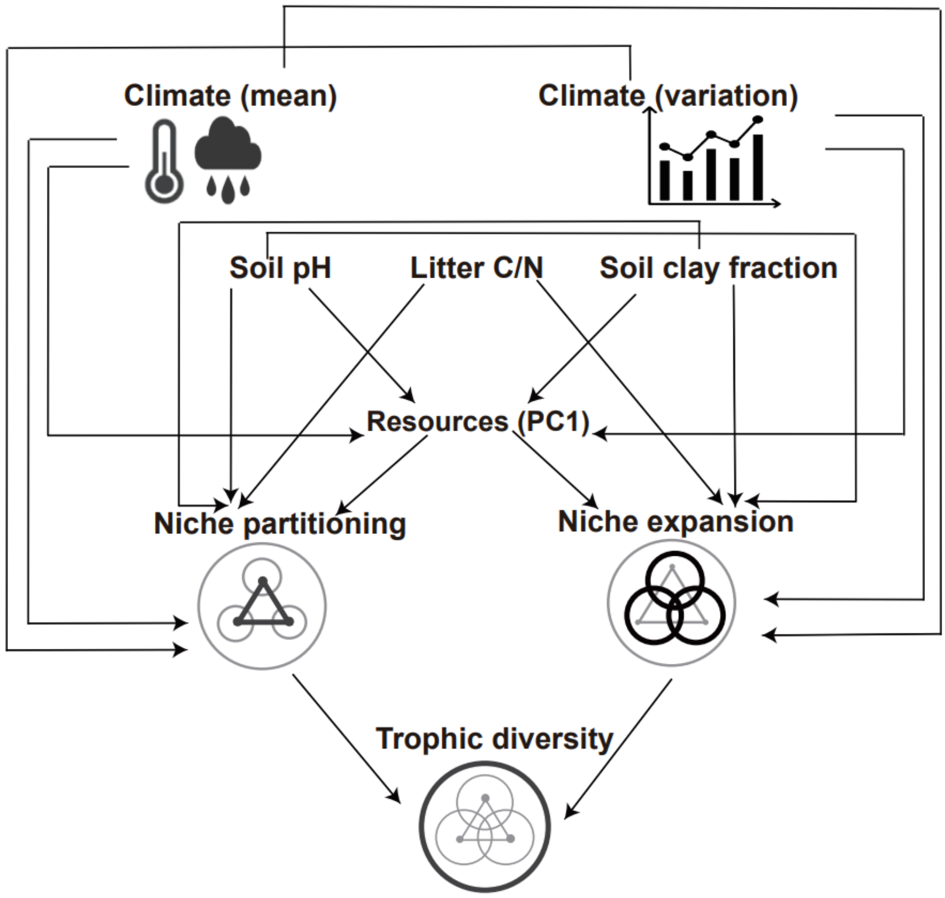

Fig. S12. Hypothesized causal pathways from climatic/edaphic factors directly to the niche partitioning/expansion or via resources.

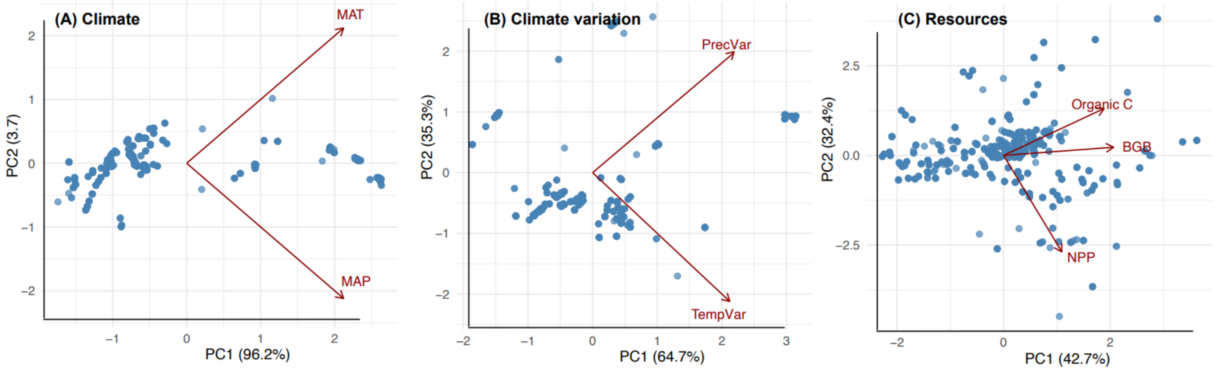

Fig. S13. PCA of Climate (A), climate variation (B) and resources (C) across study sites. MAT annual mean temperature (°C); MAP for annual mean precipitation (mm); TemVar for temperature variation of seasonality; PreVar for precipitation variation of seasonality; Organic C for soil organic C (g C soil kg^{-1}); BGB for belowground biomass (kg C m^{-2}); NPP for net primary productivity (g C m^{-2}).

Table S1 Results of linear mixed-effects model on the effects of functional group, climate (tropical vs. temperate), land use (agricultural vs. woodland), and their interactions on trophic diversity of functional groups (log-transformed SEAc at the functional-group level). SiteID and group composition was included as a random effect. Significant effects are highlighted as follows: (*) p < 0.1, * p < 0.05, ** p < 0.01, *** p < 0.001.

	**NumDF**	**DenDF**	**F**	**P-value**
Functional_group	4	72.2	4.71	0.002
LandUse	1	199.0	11.96	<0.001
Climate	1	204.9	8.85	0.003
Functional_group:LandUse	4	189.0	1.50	0.203
Functional_group:Climate	4	311.2	1.93	0.105
LandUse:Climate	1	204.4	0.58	0.448
Functional_group:LandUse:Climate	4	291.6	2.62	0.035

Table S2 Results of linear mixed-effects model on the effects of functional group, climate (tropical vs. temperate), land use (agricultural vs. woodland), and their interactions on trophic diversity of taxonomic groups (log-transformed SEAc at the high-rank taxonomic level). SiteID and taxonomic group included as a random effect. Significant effects are highlighted as follows: (*) p < 0.1, * p < 0.05, ** p < 0.01, *** p < 0.001.

	**NumDF**	**DenDF**	**F**	**P-value**
Functional_group	4	29.4	7.96	<0.001
LandUse	3	518.4	3.00	0.030
Climate	4	426.1	2.79	0.026
Functional_group:LandUse	10	502.1	1.20	0.290
Functional_group:Climate	10	621.2	2.45	0.007
LandUse:Climate	1	368.2	2.03	0.155
Functional_group:LandUse:Climate	1	600.2	7.08	0.008

Table S3 Results of linear mixed-effects model on the effects of functional group, climate (tropical vs. temperate), land use (agricultural vs. woodland), and their interactions on between-taxon trophic dissimilarity. SiteID included as a random effect. Significant effects are highlighted as follows: (*) p < 0.1, * p < 0.05, ** p < 0.01, *** p < 0.001.

	**NumDF**	**DenDF**	**F**	**P-value**
Functional_group	4	1517.9	12.02	<0.001
LandUse	1	58.4	6.21	0.016
Climate	1	88.1	19.08	<0.001
Functional_group:LandUse	4	1500.9	1.99	0.094
Functional_group:Climate	4	1515.1	2.31	0.056
LandUse:Climate	1	140.1	1.87	0.174
Functional_group:LandUse:Climate	4	1504.6	1.98	0.096

Table S4 Results of linear mixed-effects model on the effects of functional group, climate (tropical vs. temperate), land use (agricultural vs. woodland), and their interactions on the variation of ^{13}C and ^{15}N (indicated by standard deviation of ^{13}C and ^{15}N), respectively. SiteID included as a random effect.

Variation of δ^{13}C

	**NumDF**	**DenDF**	**F**	**P-value**
Functional_group	4	469.58	20.17	<0.001
LandUse	4	291.38	6.75	<0.001
Climate	3	278.43	5.30	0.001
Functional_group:LandUse	10	455.04	2.37	0.010
Functional_group:Climate	11	471.28	2.94	<0.001
LandUse:Climate	1	174.56	0.38	0.334
Functional_group:LandUse:Climate	4	416.88	6.06	<0.001

Variation of δ^{15}N

	**NumDF**	**DenDF**	**F**	**P-value**
Functional_group	4	491.47	27.36	<0.001
LandUse	4	336.63	2.58	0.076
Climate	3	323.04	17.96	<0.001
Functional_group:LandUse	10	479.43	0.73	0.816
Functional_group:Climate	11	492.35	2.43	0.012
LandUse:Climate	1	216.19	0.30	0.766
Functional_group:LandUse:Climate	4	442.74	1.35	0.289

Table S5 Results of four linear mixed-effects models on the effects of between-taxon trophic dissimilarity, taxonomic-level trophic diversity, variation of ^{13}C and ^{15}N, and their interactions with functional group on trophic diversity of functional groups (log-transformed SEAc at the functional-group level). SiteID included as a random effect.

SEAc_FG ~ MPD*Functional_group

	**NumDF**	**DenDF**	**F**	**P-value**
MPD	1	368.69	100.87	<0.001
Functional_group	4	352.57	26.78	<0.001
MPD:Functional_group	4	354.78	10.26	<0.001

SEAc_FG ~ SEAc_taxa*Functional_group

	**NumDF**	**DenDF**	**F**	**P-value**
SEAc_taxa	1	529.63	564.33	<0.001
Functional_group	4	475.65	15.15	<0.001
SEAc_taxa:Functional_group	4	489.04	24.82	<0.001

SEAc_FG ~ Functional_group*sd_d13C

	**NumDF**	**DenDF**	**F**	**P-value**
Functional_group	4	581.2	6.50	<0.001
sd_d13C	1	653.5	620.76	<0.001
Functional_group:sd_d13C	4	580.1	5.83	<0.001

SEAc_FG ~ Functional_group*sd_d15N

	**NumDF**	**DenDF**	**F**	**P-value**
Functional_group	4	647.1	7.34	<0.001
sd_d15N	1	631.8	710.13	<0.001
Functional_group:sd_d15N	4	652.0	4.81	<0.001

Table S6 Effect sizes of climate (tropical vs. temperate), land use (agricultural vs. woodland) on the trophic diversity of each functional group and across functional groups.

Contrast	group	estimate	SE	df	t.ratio	p.value	CI
Tro_Tem	Overall	0.406	0.123	173.0	3.30	<0.001	0.49
Tro_Tem	Herbivores	0.498	0.323	459.1	1.54	0.124	1.27
Tro_Tem	Detritivores	0.614	0.181	501.8	3.39	<0.001	0.71
Tro_Tem	Microbivores	0.411	0.166	495.8	2.48	0.014	0.65
Tro_Tem	Predators	0.686	0.171	494.9	4.01	<0.001	0.67
Tro_Tem	Mixed_feeders	0.177	0.246	489.8	0.72	0.472	0.97
Agr_Woo	Overall	0.321	0.110	200.2	2.93	0.004	0.43
Agr_Woo	Herbivores	-0.188	0.323	459.1	-0.58	0.561	1.27
Agr_Woo	Detritivores	0.363	0.181	501.8	2.01	0.045	0.71
Agr_Woo	Microbivores	0.578	0.166	495.8	3.49	<0.001	0.65
Agr_Woo	Predators	0.632	0.171	494.9	3.69	<0.001	0.67
Agr_Woo	Mixed_feeders	0.323	0.246	489.8	1.32	0.189	0.97

Table S7 Results of linear mixed-effects model on the effects of family richness, sampling size, and
climate and land use and their interactions with functional groups on trophic diversity of functional groups (log-transformed SEAc at the functional-group level). SiteID and group composition was included as a random effect. Significant effects are highlighted as follows: (*) $p < 0.1$, * $p < 0.05$, ** $p < 0.01$, *** $p < 0.001$.

	NumDF	DenDF	F	P-value
Functional_group	4	85.7	2.72	0.035
Climate	1	187.65	7.78	0.006
LandUse	1	228.69	9.11	0.003
Sampling size	1	346.26	0.18	0.672
Family richness	1	424.87	25.49	<0.001
Functional_group:Climate	4	204.3	6.75	<0.001
Functional_group:LandUse	4	314.88	4.40	0.002

Table S8 Results of three linear mixed-effects models on the effects of functional group, climate (tropical vs. temperate), land use (agricultural vs. woodland), and their interactions on trophic diversity of three different taxonomic levels (family, genus and species). SiteID and group composition was included as a random effect.

Family level

	NumDF	DenDF	F	P-value
Functional_group	4	538.75	3.02	0.018
Climate	1	584.3	17.14	<0.001
LandUse	1	509.8	10.37	0.001
Functional_group:Climate	4	540.02	1.41	0.230
Functional_group:LandUse	4	506.07	0.51	0.728

Genus level

	NumDF	DenDF	F	P-value
Functional_group	4	440.45	0.91	0.456
Climate	1	450.76	7.19	0.008
LandUse	1	436.69	4.12	0.043
Functional_group:Climate	3	459.9	0.19	0.904
Functional_group:LandUse	4	445.01	3.78	0.005

Species level

	NumDF	DenDF	F	P-value
Functional_group	4	335.68	0.53	0.717
Climate	1	92.25	8.51	<0.001
LandUse	1	162.11	11.52	0.001
Functional_group:Climate	2	352.3	1.99	0.138
Functional_group:LandUse	3	341.42	0.96	0.410

Table S9 P-values of environmental drivers of trophic diversity among functional groups indicated by random forests. The environmental variables of the environmental variables of NPP denotes net primary productivity (g C m-2); Organic C denotes soil organic carbon (g C soil kg-1); TemVar denotes temperature variation of seasonality; MAT denotes mean annual temperature (°C); MAP denotes mean annual precipitation (mm); AGB denotes aboveground biomass (kg C m-2), PreVar denotes precipitation variation of seasonality; BGB denotes belowground biomass (kg C m-2); clay denotes soil clay fractions (%), litter C/N denotes litter carbon and nitrogen ratio; moisture denotes soil moisture (%).

	Herbivores	Detritivores	Microbivores	Predators	Mixed feeders
NPP_gC_m2	0.010	0.040	0.010	0.010	0.010
OC_g_per_kg	0.069	0.119	0.010	0.010	0.059
pH	0.218	0.069	0.040	0.010	0.129
TempSeasonality	0.168	0.149	0.050	0.119	0.099
MAT_C	0.347	0.158	0.218	0.059	0.079
MAP_mm	0.426	0.188	0.208	0.040	0.089
AGB_kgC_m2	0.475	0.327	0.545	0.109	0.208
PrecSeasonality	0.347	0.257	0.733	0.149	0.297
BGB_kgC_m2	0.436	0.139	0.545	0.158	0.178
Clay_frac	0.614	0.386	0.525	0.416	0.238
LitterCN	0.505	0.248	0.693	0.198	0.465
water_con	0.604	0.396	1.000	0.356	0.545

Table S10 Results of pathways of environmental drivers working on trophic diversity via niche partitioning (trophic diversity of taxa) and niche expansion (trophic dissimilarity among taxa) indicated by piecewise structural equation models (piecewiseSEM). The responses include resource_PC1 with combining net primary productivity (NPP), belowground biomass and soil organic C for resources, niche partitioning, niche expansion, and trophic diversity. Predictors including climate_PC1 with combining temperature and precipitation for climate, cliVar_PC1 with combining temperature variation of seasonality and precipitation variation of seasonality for climate variation, other environmental predictors suggested in legend of Table S9. Standard estimate of predictor to response has been given in Std.Estimate.

Response	Predictor	Std.Estimate	P-Value
resource_PC1	pH	-0.6331	<0.001
resource_PC1	climate_PC1	-0.2015	0.001
resource_PC1	cliVar_PC1	-0.1962	<0.001
resource_PC1	LitterCN	0.0591	0.019
resource_PC1	Clay_frac	0.4106	<0.001
SEAc_taxa	pH	-0.0179	0.827
SEAc_taxa	climate_PC1	0.4881	<0.001
SEAc_taxa	resource_PC1	-0.1285	0.123
SEAc_taxa	cliVar_PC1	0.2223	0.003
SEAc_taxa	Clay_frac	-0.0751	0.473
SEAc_taxa	distance_each_other	0.145	0.004
SEAc_taxa	LitterCN	0.018	0.754
distance_each_other	pH	-0.1147	0.102
distance_each_other	climate_PC1	0.2121	0.040
distance_each_other	resource_PC1	-0.142	0.040
distance_each_other	cliVar_PC1	0.0862	0.185
distance_each_other	Clay_frac	0.1625	0.069
SEAc_FG	SEAc_taxa	0.6643	<0.001
SEAc_FG	distance_each_other	0.3108	<0.001
SEAc_FG	climate_PC1	0.1817	<0.001
SEAc_FG	cliVar_PC1	0.0624	0.097
SEAc_FG	pH	0.0627	0.036
