# Supplementary material for: Greater trophic diversity of soil animal communities under agricultural land use and tropical climate
Source: Nat Ecol Evol. 2026 Mar 16;10(4):700–11. doi: 10.1038/s41559-026-03014-4 (PMC13076220; doi:10.1038/s41559-026-03014-4)
Supplement: Supplementary file 2 — Reporting Summary [file 41559_2026_3014_MOESM2_ESM.pdf]

nature portfolio

Corresponding author(s): Zheng Zhou

Last updated by author(s): Nov 17, 2024

Reporting Summary

Nature Portfolio wishes to improve the reproducibility of the work that we publish. This form provides structure for consistency and transparency in reporting. For further information on Nature Portfolio policies, see our Editorial Policies and the Editorial Policy Checklist.

Statistics

For all statistical analyses, confirm that the following items are present in the figure legend, table legend, main text, or Methods section.

n/a	Confirmed	
☐	☒	The exact sample size (*n*) for each experimental group/condition, given as a discrete number and unit of measurement
☐	☒	A statement on whether measurements were taken from distinct samples or whether the same sample was measured repeatedly
☐	☒	The statistical test(s) used AND whether they are one- or two-sided<br>*Only common tests should be described solely by name; describe more complex techniques in the Methods section.*
☐	☒	A description of all covariates tested
☐	☒	A description of any assumptions or corrections, such as tests of normality and adjustment for multiple comparisons
☐	☒	A full description of the statistical parameters including central tendency (e.g. means) or other basic estimates (e.g. regression coefficient) AND variation (e.g. standard deviation) or associated estimates of uncertainty (e.g. confidence intervals)
☐	☒	For null hypothesis testing, the test statistic (e.g. *F*, *t*, *r*) with confidence intervals, effect sizes, degrees of freedom and *P* value noted<br>*Give P values as exact values whenever suitable.*
☒	☐	For Bayesian analysis, information on the choice of priors and Markov chain Monte Carlo settings
☒	☐	For hierarchical and complex designs, identification of the appropriate level for tests and full reporting of outcomes
☒	☐	Estimates of effect sizes (e.g. Cohen's *d*, Pearson's *r*), indicating how they were calculated

Our web collection on statistics for biologists contains articles on many of the points above.

Software and code

Policy information about availability of computer code

Data collection	no software was used for collecting the data
Data analysis	Analysis was implemented in R v4.2.0 with R studio interface v1.4.1103 (RStudio, PBC). The following packages were used: SIBER v2.1.9, ggpubr v0.6.0, maps v3.4.1, vegan v2.6-8, ggplot2 v3.5.1, nlme v3.1-162, effects v4.2-2, dplyr v1.1.4, tidyr v1.3.0, readxl v1.4.2, lme4 v1.1-32, lmerTest v3.1-3, emmeans v1.10.4. Linear models are specified in the Extended Data Table 1-5, 7 and 8. The code for figures and statistics are available here in figshare: https://figshare.com/s/c4a378183d4d35e982d1

For manuscripts utilizing custom algorithms or software that are central to the research but not yet described in published literature, software must be made available to editors and reviewers. We strongly encourage code deposition in a community repository (e.g. GitHub). See the Nature Portfolio guidelines for submitting code & software for further information.

Data

Policy information about availability of data

All manuscripts must include a data availability statement. This statement should provide the following information, where applicable:

- Accession codes, unique identifiers, or web links for publicly available datasets
- A description of any restrictions on data availability
- For clinical datasets or third party data, please ensure that the statement adheres to our policy

Data are avaiable here: https://figshare.com/s/c4a378183d4d35e982d1

Research involving human participants, their data, or biological material

Policy information about studies with human participants or human data. See also policy information about sex, gender (identity/presentation), and sexual orientation and race, ethnicity and racism.

Reporting on sex and gender	N/A
Reporting on race, ethnicity, or other socially relevant groupings	N/A
Population characteristics	N/A
Recruitment	N/A
Ethics oversight	N/A

Note that full information on the approval of the study protocol must also be provided in the manuscript.

Field-specific reporting

Please select the one below that is the best fit for your research. If you are not sure, read the appropriate sections before making your selection.

☐ Life sciences ☐ Behavioural & social sciences ☒ Ecological, evolutionary & environmental sciences

For a reference copy of the document with all sections, see nature.com/documents/nr-reporting-summary-flat.pdf

Ecological, evolutionary & environmental sciences study design

All studies must disclose on these points even when the disclosure is negative.

Study description	The study explores the trophic diversity of soil animals between different functional groups, land-use and climatic systems.
Research sample	The study analyses 26 high-rank taxonomic groups of soil animals (including earthworms, nematodes, spiders, etc)
Sampling strategy	The dataset comprised 15,893 sample records of paired δ13C and δ15N values in soil animals across 343 study sites and 15 countries, The investigated ecosystem types included woodlands, agricultural systems and grasslands.
Data collection	Authors of the paper collected the soil animals and conducted the stable isotope analysis.
Timing and spatial scale	Samples distributed across four climatic regions: subarctic, temperate, subtropical, and tropical regions, and from 2002 - 2018.
Data exclusions	No data were excluded from the analysis
Reproducibility	R code and statistical model specifications are openly available allowing to reproduce the data analysis: https://figshare.com/s/c4a378183d4d35e982d1
Randomization	In this study, randomization was not applicable as the data were derived from a combination of published datasets and field sampling. Data collection followed standardized methods, with soil animals sampled systematically from the litter layer and topsoil using established protocols, ensuring consistency across locations. Further details of the sampling methods and sites are provided in Table S8.
Blinding	Blinding was not relevant to this study as it involved ecological data collected across multiple geographic locations and climatic regions. Data analysis was conducted objectively using numerical values (e.g., stable isotope ratios, climatic variables) without subjective interpretation that would necessitate blinding.

Did the study involve field work? ☒ Yes ☐ No

Field work, collection and transport

Field conditions	Fieldwork was conducted across 343 sites spanning subarctic, temperate, subtropical, and tropical regions. Key abiotic parameters, including mean annual precipitation and temperature, were recorded using WorldClim data based on site coordinates. The details of study sites are listed in Table S8.
Location	Sampling locations were distributed across 15 countries, with precise latitude and longitude details for each site listed in Table S8.

Access & import/export	All sampling was conducted in compliance with local, national, and international regulations. Necessary permits for sampling, transportation, and export of samples were obtained where required.
Disturbance	Work on the study sites was implemented with care, to minimize disturbance. Whenever possible, manipulations with samples were done in a laboratory, outside the field sampling areas.

Reporting for specific materials, systems and methods

We require information from authors about some types of materials, experimental systems and methods used in many studies. Here, indicate whether each material, system or method listed is relevant to your study. If you are not sure if a list item applies to your research, read the appropriate section before selecting a response.

Materials & experimental systems

n/a	Involved in the study
☒	☐ Antibodies
☒	☐ Eukaryotic cell lines
☒	☐ Palaeontology and archaeology
☐	☒ Animals and other organisms
☒	☐ Clinical data
☒	☐ Dual use research of concern
☒	☐ Plants

Methods

n/a	Involved in the study
☒	☐ ChIP-seq
☒	☐ Flow cytometry
☒	☐ MRI-based neuroimaging

Animals and other research organisms

Policy information about studies involving animals; ARRIVE guidelines recommended for reporting animal research, and Sex and Gender in Research

Laboratory animals	Study did not involve laboratory animals
Wild animals	Only soil invertebrate animals (arthropods and earthworms) were collected and killed using ethanol during the study. This was necessary to assess stable isotope composition. We collected soil arthropod and earthworm communities using Kempson extractors.
Reporting on sex	Sex was not considered in the study
Field-collected samples	Collected soil samples were transported in the lab for heat extraction. No field-collected environmental samples were used in this study.
Ethics oversight	No ethical approval was required. The study did not involve vertebrate animal capturing and killing.

Note that full information on the approval of the study protocol must also be provided in the manuscript.

Plants

Seed stocks	N/A
Novel plant genotypes	N/A
Authentication	N/A
